# The cognitive effect of non-invasive brain stimulation combined with cognitive training in Alzheimer’s disease and mild cognitive impairment: a systematic review and meta-analysis

**DOI:** 10.1186/s13195-024-01505-9

**Published:** 2024-06-27

**Authors:** Ting Yang, Wentao Liu, Jiali He, Chenfan Gui, Lijiao Meng, Li Xu, Chengsen Jia

**Affiliations:** 1 Department of Rehabilitation Medicine, West China Tianfu Hospital, Sichuan University, No. 3966, South Section 2, Tianfu Avenue, Tianfu New Area, Chengdu, 610212 Sichuan China; 2 Department of Rehabilitation Medicine Center, West China Hospital, Sichuan University, No. 37, Guo Xue Alley, Chengdu, 610041 Sichuan China; 3 Key Laboratory of Rehabilitation Medicine in Sichuan Province, West China Hospital, Sichuan University, No. 37, Guo Xue Alley, Chengdu, 610041 Sichuan China; 4 Department of Rehabilitation Medicine, The Second Hospital of Chongzhou, No. 431, Tang’an West Road, Chongyang Town, Chongzhou City, Chengdu, 611230 Sichuan China

**Keywords:** Non-invasive brain stimulation, Cognitive training, Alzheimer’s disease, Mild cognitive impairment, Repetitive transcranial magnetic stimulation, Transcranial direct current stimulation, Cognitive function

## Abstract

**Background:**

Non-invasive brain stimulation (NIBS) combined with cognitive training (CT) may have shown some prospects on improving cognitive function in patients with Alzheimer’s disease (AD) and mild cognitive impairment (MCI). However, data from clinical trials or meta-analysis involving NIBS combined with CT have shown controversial results. The aim of this systematic review and meta-analysis was to evaluate short-term and long-term effects of NIBS combined with CT on improving global cognition and other specific cognitive domains in patients with AD and MCI.

**Methods:**

This systematic review and meta-analysis was conducted in accordance with the Preferred Reporting Items for Systematic Reviews and Meta-Analyses (PRISMA) guidelines. Five electronic databases including PubMed, Web of Science, EBSCO, Cochrane Library and Embase were searched up from inception to 20 November 2023. The PEDro scale and the Cochrane’s risk of bias assessment were used to evaluate risk of bias and methodological quality of included studies. All statistical analyses were conducted with Review Manager 5.3.

**Results:**

We included 15 studies with 685 patients. The PEDro scale was used to assess methodological quality with a mean score of 7.9. The results of meta-analysis showed that NIBS combined with CT was effective on improving global cognition in AD and MCI (SMD = 0.52, 95% CI (0.18, 0.87), *p* = 0.003), especially for patients accepting repetitive transcranial magnetic stimulation (rTMS) combined with CT (SMD = 0.46, 95% CI (0.14, 0.78), *p* = 0.005). AD could achieve global cognition improvement from NIBS combined with CT group (SMD = 0.77, 95% CI (0.19, 1.35), *p* = 0.01). Transcranial direct current stimulation (tDCS) combined with CT could improve language function in AD and MCI (SMD = 0.29, 95% CI (0.03, 0.55), *p* = 0.03). At evaluation follow-up, rTMS combined with CT exhibited larger therapeutic responses to AD and MCI in global cognition (SMD = 0.55, 95% CI (0.09, 1.02), *p* = 0.02). AD could achieve global cognition (SMD = 0.40, 95% CI (0.03, 0.77), *p* = 0.03) and attention/working memory (SMD = 0.72, 95% CI (0.23, 1.20), *p* = 0.004) improvement after evaluation follow-up from NIBS combined with CT group.

**Conclusions:**

Overall, NIBS combined with CT, particularly rTMS combined with CT, has both short-term and follow-up effects on improving global cognition, mainly in patients with AD. tDCS combined with CT has advantages on improving language function in AD and MCI. Future more studies need evaluate cognitive effects of NIBS combined with CT on other specific cognitive domain in patients with cognitive deterioration.

**Supplementary Information:**

The online version contains supplementary material available at 10.1186/s13195-024-01505-9.

## Introduction

Alzheimer’s disease (AD) is the most common neurodegenerative disease with severe deterioration of cognitive function and activity of daily living [1]. Mild cognitive impairment (MCI) is the preclinical stage of AD and every patient who develops AD would first experiences this stage [2]. In China, epidemiological investigations show that the estimated prevalence of MCI is 15.5% among adults aged over 60 years [3]. Among those with MCI, about 15% would develop dementia after 2 years, and 33% progress to AD within 5 years [4, 5]. Progressive cognitive deterioration imposes a heavy burden on patients and their families. The economic value of care to be provided by families and other unpaid caregivers of patients with dementia has reached $339.5 billion in the United States in 2022 [1], meanwhile, the cost of social care for AD is higher than the global average in China [6]. While some pharmacological interventions, such as monoclonal antibodies targeting Aβ (e.g., Lecanemab) [7], have demonstrated potential benefits in mitigating cognitive decline and preserving function in early AD, the overall effectiveness of these treatments remains limited and warrants further investigation [8]. In recent years, there is growing interest in exploring the benefits of non-pharmacological interventions.

Non-invasive brain stimulation (NIBS), typically including repetitive transcranial magnetic stimulation (rTMS) and transcranial direct current stimulation (tDCS), is a class of cost-effective, safe, and easy-to-administer techniques which can modulate brain excitability and plasticity to improve cognition function in AD and MCI [9, 10]. However, a meta-analysis by Inagawa et al. [11] thought NIBS showed limited effects on improving cognitive function in AD and MCI. Cognitive training (CT) is defined as treatment focusing on guided practice on tasks for specific cognitive functions. Plenty of evidences indicated that CT could improve cognitive functions in AD and MCI [12–15], possibly due to the reciprocity between cognitive mental activity stimulated by CT and cerebral biochemistry [16]. NIBS modulates neural plasticity directly in targeted regions and networks of brain, while CT may improve cognitive function in AD and MCI by indirectly modulate brain plasticity. A randomized controlled trials by Lee et al. [17] found a significant effect of rTMS combined with CT on improving memory and language domains in AD. Similarly, another clinical trial by Andrade et al. [18] showed tDCS combined with CT modulated cortical activity and improved global cognition in AD. NIBS combined with CT for AD and MCI seems to achieve better cognitive improvement, however, there is still a lack of high-level evidence at present.

Current research on the effects of NIBS combined with CT on improving cognitive function has shown controversial results. Two meta-analyses results found NIBS combined with CT had no conclusive advantage on improving cognitive function in MCI or AD [9, 19]. Those meta-analyses included few studies to qualitative synthesis, and the overall certainty of evidence was very low. Another meta-analysis including patients with Parkinson’s disease, MCI, AD and other multiple neuropsychiatric disorders [20], but the result did not find the effects of NIBS combined with CT. That meta-analysis might result in high heterogeneity due to different types of patients included. Consequently, we completed a systematic review and meta-analysis to re-evaluate the effect of NIBS combined with CT on cognitive function in AD and MCI from all available clinical studies when compared to only NIBS, CT or placebo. This will help us better understand the potential of NIBS combined with CT to provide solutions for cognitive deterioration, with the aim of outlining more robust interventions for patients with AD and MCI in the future.

## Methods

This systematic review and meta-analysis was conducted in accordance with the Preferred Reporting Items for Systematic Reviews and Meta-Analyses (PRISMA) guidelines [21]. The protocol of this review was registered in the International Prospective Register of Systematic Reviews (PROSPERO): CRD42023417926.

### Search strategy

The search from the earliest available to 20 November 2023 was identified in following databases: PubMed, Web of Science, EBSCO, Cochrane Library and Embase. The selected keywords and search strategy were shown in supplementary material 1. Hand searching was also conducted to identify potentially relevant studies.

### Eligibility criteria

The inclusion criteria were determined according to the PICOS approach: (1) patients were diagnosed with MCI or AD according to Peterson`s criteria of MCI [22], DSM-5 [23] or NIA-AA`s criteria of AD [24]; (2) the interventions were combination of NIBS(e.g., tDCS or rTMS) with CT; (3) the control group could be either a combination of CT with sham NIBS, a combination of NIBS with sham CT, only CT, only NIBS, or a placebo group; (4) study design was randomized controlled trial (RCT) or randomized cross-over design published; (5) articles were published in English. The exclusion criteria were as follow: (1) other intervention than NIBS or CT; (2) participants aged < 60 years; (3) studies were published as conference proceedings or dissertations.

### Data extraction and quality assessment

The included studies were independently reviewed and selected based on the eligibility criteria by two reviewers (WL and CG). Titles and abstracts of all potentially relevant studies were screened, and full texts of the possible included studies were then screened for final inclusion. Another two reviewers (TY and JH) extracted required data of all included studies independently into a predesigned sheet. The data extracted from those studies included first author, year of publication, study characteristics (study design, population, intervention time, group design, NIBS parameters and follow-up time) and outcome measures. Corresponding authors of included records were contacted for missing data. Primary articles with missing data/variables that could not be used for all outcomes analyses were not included in this review. Any disagreements during data extraction were discussed and adjudicated by a third reviewer (LM).

Methodological quality assessment for each study was assessed using items adapted from the PEDro scale [25]. Two experienced reviewers (TY and WL) independently rated the included studies using the PEDro scale. Risk of bias assessments for each study were conducted by two experienced reviewers (TY and WL) according to the criteria in the *Cochrane Handbook for Systematic Reviews of Interventions *[26]. These items were designed to assess whether the study contained methodological bias that could affect meta-analysis results. When any disagreements during the assessments were discussed, a third reviewer (LX) participated in negotiation to jointly decide the quality of the included studies.

### Data analysis

The results of all included RCTs and cross-over designs studies were used standard meta-analytic methods to evaluate the effects of NIBS combined with CT in AD and MCI. The means and standard deviations (SDs) of the change were used to calculate the absolute magnitude of change of outcome measures after interventions for experiment and control groups. The standardised mean differences (SMDs) with 95% confidence intervals (CIs) were calculated for continuous variables. Significant difference was set as *P*-value ≤ 0.05, and 95% CIs were also presented. Statistical heterogeneity was evaluated using chi-square test and I^*2*^ statistic. The values of I^2^ > 40% was considered to represent high statistical heterogeneity [27]. All meta-analysis results were performed using a random effects model, because there could be variability between studies due to different diagnostic types or applications of NIBS interventions. In this review, we chose to conduct separate meta-analysis for any cognitive domain that were investigated in at least 3 included studies. All statistical analysis was conducted using Review Manager 5.3.

## Results

### Search results

**Fig. 1:**
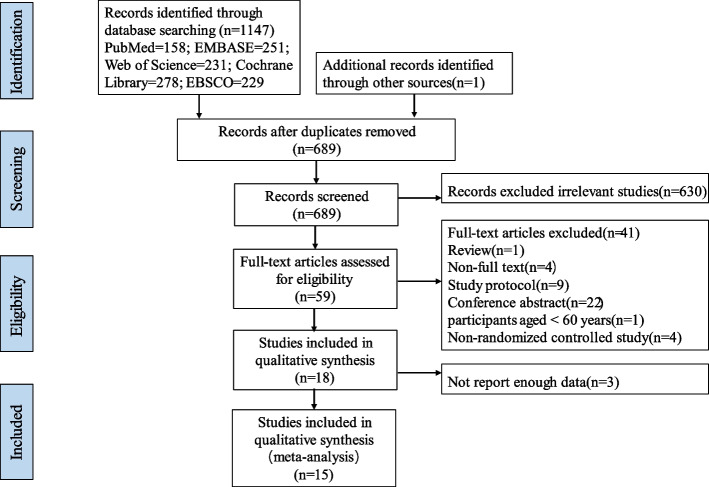
PRISMA flow diagram

According to before mentioned search strategy, 1148 published studies were identifies from the selected database. Fifty-nine studies were retrieved after screening titles and abstracts. Forty-one studies were excluded due to study design (*n* = 37; 1 review, 9 study protocols, 22 conference abstracts, 1 participants aged < 60 years, 4 non-randomized controlled studies), full texts not available (*n* = 4). Three additional studies were excluded as complete data was not obtained from the articles or authors. Finally, 15 studies with 685 patients met the eligibility criteria (Fig. 1). Patients demographic characteristics were found in Table 1. Mean age of patients included studies ranged between 69.0 and 76.6 years old, and education years of most patients had mean over 6 years except 2 studies [18, 28]. For pre-treatment cognitive assessment, Lu et al. [29] used ADAS-Cog, Gonzalez et al. [30] used MoCA, and the others used MMSE.

**Table 1:** Patients demographic characteristics of included studies in this review

	**Age (years)**	**Gender (M/F)**	**Education (years)**	**Baseline cognition:** **MMSE/MoCA/ADAS-Cog**	**Age (years)**	**Gender (M/F)**	**Education (years)**	**Baseline cognition:** **MMSE/MoCA/ADAS-Cog**
**References**	**Experiment group**				**Control group**			
Rodella et al. (2021) [31]	71.62 ± 5.65	8/5	11.08 ± 4.99	23.84 ± 2.99	75.13 ± 4.76	9/6	9.67 ± 4.98	22.98 ± 2.22
Martin et al. (2019) [32]	71.8 ± 6.39	13/20	14.5 ± 3.51	NA	71.6 ± 6.35	10/25	14.9 ± 3.23	NA
Andrade et al. (2022) [18]	75.4 ± 4.7	10/8	4.4 ± 2.7	20.2 ± 0.9	77.1 ± 5.2	9/9	5.6 ± 3.1	20.4 ± 1.1
Lu et al. (2019) [29]	74.2 ± 6.7	21/42	7.3 ± 4.8	9.4 ± 3.9^b^	73.9 ± 6.3	44/66	7.0 ± 4.8	9.6 ± 3.9^b^
Gonzalez et al. (2021) [30]	69.8 ± 5.3	6/15	9.7 ± 3.6	23.7 ± 1.7^a^	70.8 ± 5.8	12/33	10.7 ± 4.4	24.2 ± 2.1^a^
Inagawa et al. (2019) [33]	76.6 ± 5.7	3/4	NA	NA	76.2 ± 7.7	7/6	NA	NA
Roncero et al. (2017) [34]	NA	NA	NA	NA	NA	NA	NA	NA
de Sousa et al. (2020) [35]	NA	NA	NA	NA	NA	NA	NA	NA
Cotelli et al. (2014) [28]	76.6 ± 4.6	2/10	5.5 ± 2.4	20.1 ± 2.4	74.7 ± 6.1	3/9	8.9 ± 5.1	20.8 ± 2.1
Brem et al. (2020) [36]	69.25 ± 6.80	4/12	14.25 ± 4.64	21.19 ± 2.69	68.39 ± 7.66	10/8	15.5 ± 4.86	21.50 ± 2.38
Vecchio et al. (2022) [37]	71.07 ± 1.25	14/16	13.87 ± 0.78	22.93 ± 0.51	73.68 ± 2.71	15/18	11.63 ± 1.22	20.81 ± 0.74
Rabey et al. (2013) [38]	72.6 ± 8.9	5/2	NA	22 ± 1.63	75.4 ± 9.07	5/3	NA	22 ± 1.41
Lee et al. (2016) [17]	72.1 ± 7.6	8/10	9.9 ± 4.8	22.4 ± 2.9	70.3 ± 4.8	3/5	9.9 ± 3.7	22.8 ± 2.5
Bagattini et al. (2020) [39]	73.56 ± 4.91	17/10	8.85 ± 3.91	23.67 ± 3.00	73.35 ± 1.09	12/11	7.91 ± 0.67	22.77 ± 3.09
Zhang et al. (2019) [40]	69.00 ± 8.19	3/10	12.40 ± 2.06	20.53 ± 4.17	68.54 ± 7.93	3/12	11.85 ± 2.38	19.83 ± 5.10

Values were presented as mean ± standard deviation or numbers

*M/F* male/female, *MMSE* Mini-Mental State Examination, *MoCA* Montreal Cognitive Assessment, *ADAS-Cog* Alzheimer Disease Assessment Scale, cognitive subscale, *NA* not available

^a^Scores denoted MoCA score

^b^Scores denoted ADAS-Cog score

### Study characteristics

Details of 15 included studies were summarized in Table 2. Studies included in this meta-analysis were published between 2013 and 2022. Among those studies, 9 used tDCS as intervention of NIBS [18, 28–35], another 6 used rTMS [17, 36–40]. Two studies used randomized cross-over design [34, 35], the others used randomized controlled design. For target patients, 4 studies included MCI [29, 30, 32, 35], 9 studies included AD or other dementia [17, 18, 28, 33, 34, 36–38, 40], and 2 studies included both AD and MCI [31, 39]. For tDCS stimulation montage, anodal tDCS F3 montage [28, 30–33] was utilized in half studies, while other studies utilized anodal tDCS T3 montage [29], P3 montage [34], and T6 montage [35], respectively. Only 1 study chose multisite anodal tDCS montages including F3, F4, F5, P4, P5 and CP5 [18]. For stimulation montage of rTMS, 5 studies utilized multisite montages [17, 36–38, 40], except 1 study used F3 montage [39]. Most of studeis administered NIBS stimulation and CT simultaneously, except 1 studies administered tDCS earlier than CT [32] and 1 study administered rTMS earlier than CT [40]. We obtained follow-up data from 11 studies, while 2 studies were unable to be included in results analysis due to missing follow-up data [37, 38]. Two studies did not include follow-up assessments in their methodology [18, 35].

**Table 2:** Study characteristics in this review

**References**	**Study design**	**Population**	**Group design**	**Intervention “DOSE”**	**Stimulation montage**	**Intensity and duration**	**CT type**	**CT and NIBS timing**	**Follow-up**
Rodella et al. (2021) [31]	Double-blind, randomized, prospective	9 aMCI; 19 mid AD	tDCS + CT; sham tDCS + CT	12 sessions over 3 weeks	Anode: left DLPFC, Cathode: right deltoid muscle	2 mA, 30 min	Computerized(multi-domain CoRe software)	simultaneous	6 months
Martin et al. (2019) [32]	Double-blind, randomized, parallel	68 aMCI	tDCS + CT; sham tDCS + CT	15 sessions over 5 weeks	Anode: left DLPFC, Cathode: F8	2 mA, 30 min	Computerized(COGPACK)	no overlap (tDCS prior to CT)	3 months
Andrade et al. (2022) [18]	Double-blind, randomized	36 probable AD	tDCS + CT; sham tDCS + CT	24 sessions over 8 weeks	Anode: A (F5, CP5, F4) and B (F3, P4, P5)—10 min/area; Cathode: contralateral supraorbital area corresponding to the anode electrode	2 mA, 30 min	standardized cognitive training	simultaneous	—
Lu et al. (2019) [29]	Double-blind, randomized, parallel	201 Mild NCD-AD	tDCS + CT; sham tDCS + CT; tDCS + placebo-controlled	12 sessions over 4 weeks	Anode: T3, Cathode: contralateral upper limb	2 mA, 20 min	Computerized (Adaptive N-back test)	simultaneous	4 weeks
Gonzalez et al. (2021) [30]	Double-blind, randomized	66 MCI	tDCS + CT; sham tDCS + CT; CT only	9 sessions over 3 weeks	Anode: F3, Cathode: contralateral brachioradialis muscle	1.5 mA, 30 min	Computerized (Neuron Up)	simultaneous	6 weeks
Inagawa et al. (2019) [33]	Double-blind, randomized	16 AD and 4 other dementia	tDCS + CT; sham tDCS	10 sessions over 5 consecutive days	Anode: F3, Cathode: Fp2	2 mA, 20 min	Paper-and-pencil calculation and language tasks	simultaneous	2 weeks
Roncero et al. (2017) [34]	Double blind, randomized, cross-over	3 AD and 7 frontotemporal dementia	tDCS + CT; sham tDCS (crossover between 2 months)	10 daily sessions per crossover	Anode: P3, Cathode: right front orbital area	2 mA, 30 min	Spontaneous naming test from standardized cognitive training	simultaneous	2 weeks
de Sousa et al. (2020) [35]	Subject-blind randomized, cross-over	16 MCI	tDCS + CT; sham tDCS (crossover between 3 months)	3-day sessions per crossover	Anode: T6, Cathode: left supraorbital area	1 mA, 20 min	Computerized (visuospatial memory training)	simultaneous	—
Cotelli et al.(2014) [28]	Double-blind, randomized	24 AD	tDCS + CT; sham tDCS + CT; tDCS + motor training	10 sessions over 2 weeks	Anode: left DLPFC, Cathode: right deltoid muscle	2 mA, 25 min	Computerized(developed from patient performance on FNAT)	simultaneous	3 months
Brem et al. (2020) [36]	Double-blind, randomized, sham-controlled	34 mild-moderate AD	rTMS + CT; sham rTMS + sham CT; sham rTMS + CT	30 sessions over 6 weeks	Right/left DLPFC, right/left IPL, left STG and left IFG	Intensity: 120% RMT; Frequency: 20 trains, 2 s of 10 Hz	Computerized (NeuroAD system)	simultaneous	4–6 weeks
Vecchio et al. (2022) [37]	Double-blind, randomized, sham-controlled	63 AD	rTMS + CT; sham rTMS + CT; sham rTMS + sham CT	30 sessions over 6 weeks	Broca’s area, R-DLPFC and L-DLPFC (A group), Wernicke’s area R-pSAC and L-pSAC(B group)	Intensity:, 90% or 110% RMT; Frequency: each brain area: 20–30 trains, 2 s of 10 Hz(20 s pulses), total 1500 pulses	Computerized (NeuroAD system)	simultaneous	NA
Rabey et al. (2013) [38]	Double-blind, randomized, sham-controlled	15 mild to moderate AD	rTMS + CT; sham rTMS + CT	IP:30 sessions over 6 weeks; MP: 2 sessions/week over 3 months	Broca, R-DLPFC and L-DLPFC, Wernicke, R-pSAC and L-pSAC	Intensity: 90% RMT at Broca and DLPFC; 110% RMT at Wernicke and pSAC; Frequency: Random two regions: 20 trains, 2 s of 10 Hz(20 s pulses), total 1300 pulses	Computerized (NeuroAD system)	simultaneous	NA
Lee et al. (2016) [17]	Double-blind randomized, sham-controlled	19 mild AD; 7 moderate AD	rTMS + CT; sham rTMS + sham CT	30 sessions over 6 weeks	Right and left DLPFC, Broca’s and Wernicke’s areas, and right and left pSAC	Intensity: 90% RMT at Broca and DLPFC; 110% RMT at Wernicke and pSAC; Frequency: 20 trains, 2 s of 10 Hz(20 s pulses), total 1200 pulses	Computerized (NeuroAD system)	simultaneous	6 weeks
Bagattini et al. (2020) [39]	Double-blind, randomized, sham-controlled	50 amnesic MCI or AD	rTMS + CT; sham rTMS + CT	20 sessions over 4 weeks	Left DLPFC	Intensity: 100% RMT; Frequency: 50 trains, 2 s of 20 Hz(28 s pauses). Total 2,000 pulses	Computerized (RehabCom, Hasomed, GmbH)	simultaneous	8 weeks
Zhang et al. (2019) [40]	Double-blind, randomized, sham-controlled	28 probable AD	rTMS + CT; sham rTMS + CT	20 sessions over 4 weeks	First left DLPFC, then left LTL	Intensity: 100% RMT. Frequency: 20 trains, 5 s of 10 Hz( 25 s pauses). Total 1,000 pulses	cognitive tasks on an iPad tablet	no overlap(rTMS prior to CT)	4 weeks

*MCI* mild cognitive impairment, *AD* Alzheimer’s disease, *NCD-AD* neurocognitive disorder due to Alzheimer’s disease, *NIBS* noninvasive brain stimulation, *tDCS* transcranial direct current stimulation, *rTMS* repetitive transcranial magnetic stimulation, *CT* cognitive training, *IP* intensive phase, *MP* maintenance phase, *DLPFC* dorsolateral prefrontal cortex, *IPL* inferior parietal lobule, *IFG* inferior frontal gyrus, *STG* superior temporal gyrus, *pSAC* parietal somatosensory association cortex, *RMT* resting motor threshold, *NA* not available

### Risk of bias assessment

**Fig. 2:**
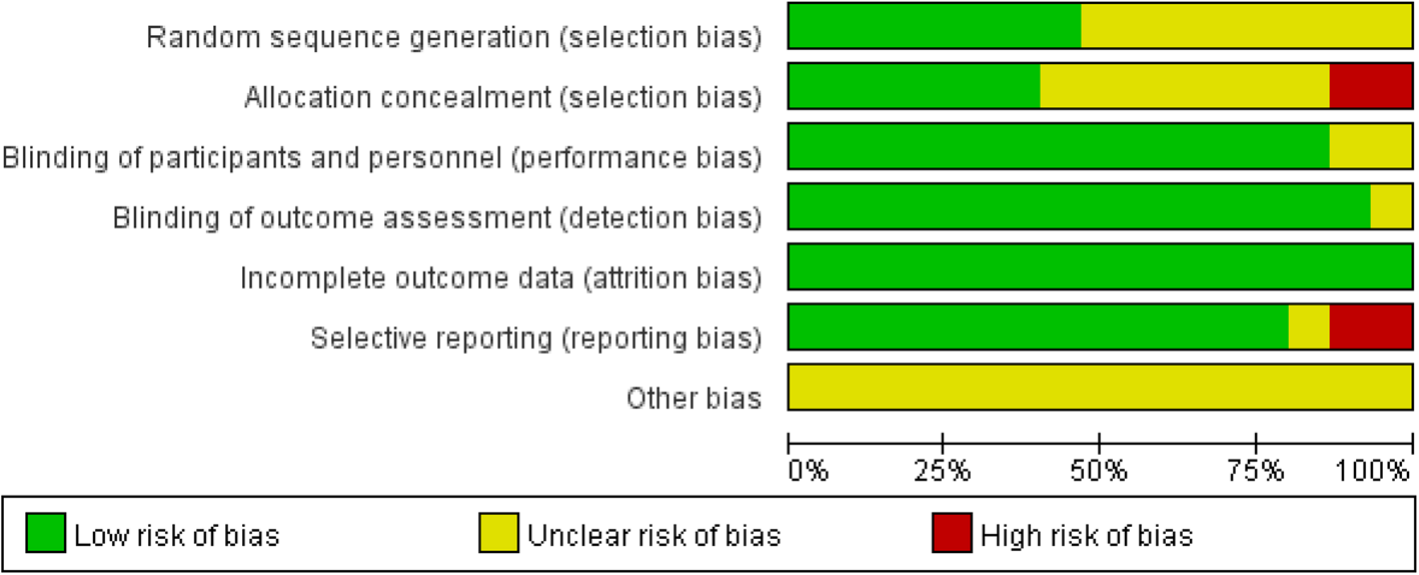
Risk of bias graph according to the Cochrane risk of bias tool

**Fig. 3:**
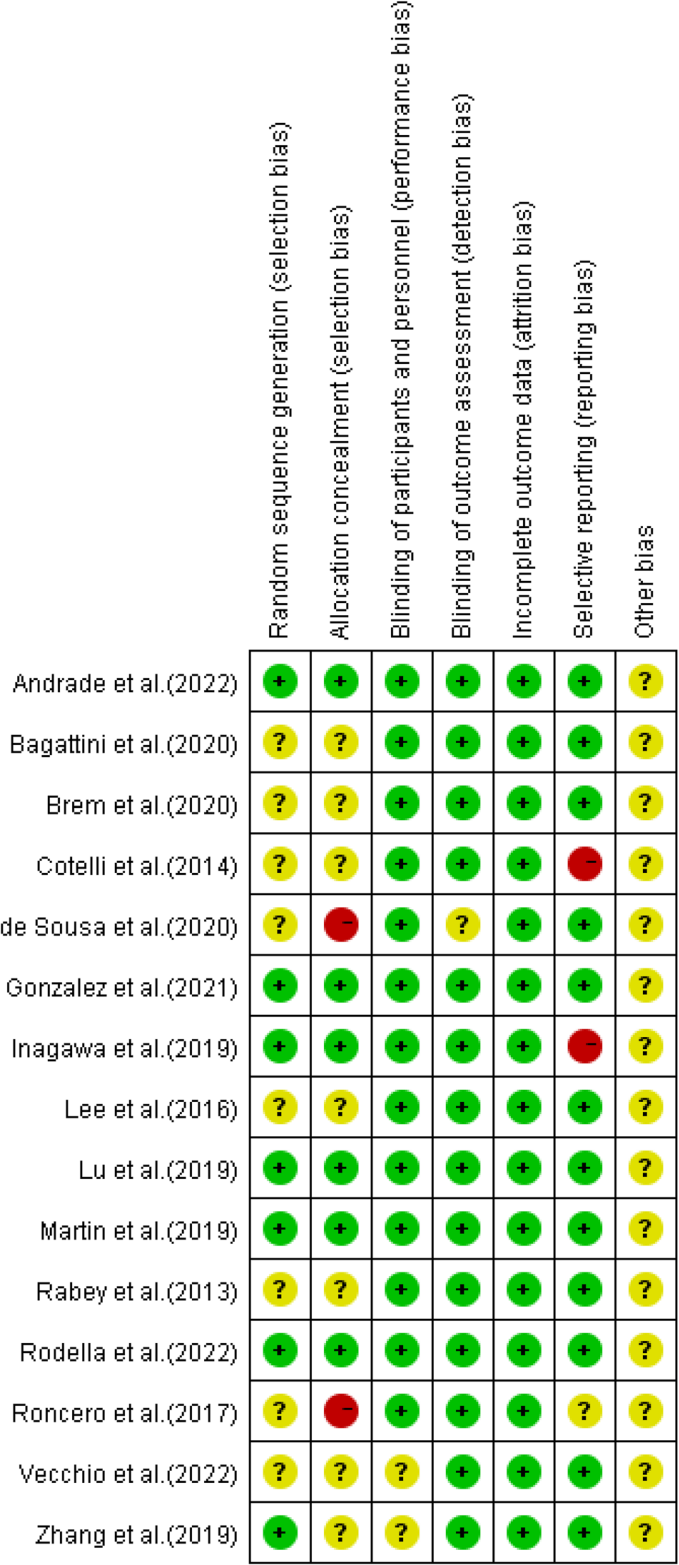
Risk of bias summary according to the Cochrane risk of bias tool: “ + ”, “-” and “?” respectively indicate low, high, and unclear risk of bias

The PEDro scores ranged from 6 to 9, with a median of 7.9, indicating that the methodological quality of included studies was relatively high. All included studies were classified with “Excellent” or “Good” quality, reporting adequately with regard to their “random allocation” and “blind subjects”. However, no studies satisfied the “blind therapists” criteria. A detailed evaluation of PEDro scores was shown in Table 3. In risk of bias assessments, 4 studies were found to have high potential risk of bias because of insufficient concealing group allocation for patients or no fully reporting primary outcomes [28, 33–35]. Risk of bias assessments with included studies in this review were shown in Figs. 2 and 3.

**Table 3:** PEDro assessment quality results in this review

**References**	**Eligibility**^**a**^	**Random allocation**	**Concealed allocation**	**Baseline comparability**	**Blind subjects**	**Blind therapists**	**Blind assessors**	**Adequate follow-up**	**Intention-to-treat analysis**	**Between- group comparisons**	**Point estimates and variability**	**Total score (0–10)**	**Quality**
Rodella et al. (2021) [31]	Yes	1	1	1	1	0	1	1	0	1	1	8	good
Martin et al. (2019) [32]	Yes	1	1	1	1	0	1	1	1	1	1	9	excellent
Andrade et al. (2022) [18]	Yes	1	1	1	1	0	1	1	1	1	1	9	excellent
Lu et al. (2019) [29]	Yes	1	1	1	1	0	1	1	1	1	1	9	excellent
Gonzalez et al. (2021) [30]	Yes	1	1	1	1	0	1	1	1	1	1	9	excellent
Inagawa et al. (2019) [33]	Yes	1	1	1	1	0	1	1	1	1	0	8	good
Roncero et al. (2017) [34]	Yes	1	0	1	1	0	1	1	0	1	1	7	good
de Sousa et al. (2020) [35]	Yes	1	0	0	1	0	1	1	0	1	1	6	good
Cotelli et al. (2014) [28]	Yes	1	1	1	1	0	1	1	1	1	1	8	good
Brem et al. (2020) [36]	Yes	1	1	1	1	0	1	1	0	1	1	8	good
Vecchio et al. (2022) [37]	Yes	1	0	1	1	0	1	1	1	1	1	8	good
Rabey et al. (2013) [38]	Yes	1	0	1	1	1	1	1	0	1	1	7	good
Lee et al. (2016) [17]	Yes	1	0	1	1	0	1	1	0	1	1	7	good
Bagattini et al. (2020) [39]	Yes	1	0	1	1	1	1	1	1	1	1	9	excellent
Zhang et al. (2019) [40]	Yes	1	0	1	1	0	1	1	0	1	1	7	good

^a^Eligibility criteria is not included in the scoring of PEDro scale

### Meta-analysis results

Due to the limited or absent data available of rTMS combined with CT studies on specific cognitive domains, we conducted separate meta-analysis for specific cognitive domain in tDCS combined with CT studies. Only subgroup analysis was performed exploring both tDCS and rTMS on global cognition. In this review, cognitive domains were analyzed including global cognition, executive function, attention/working memory, memory, and language. Cognitive domains and outcome measures for each study were shown in Table 4.

**Table 4:** Cognitive domains and outcome measures

**References**	**Primary outcome**	**Second outcome**	**Global cognition**	**Attention/working memory**	**Memory**	**Execution**	**Language**
Rodella et al. (2021) [31]	MMSE	specific cognitive domains	MMSE	Digit Span	Rey’s 15 words test delayed recall	FAB	—
Martin et al. (2019) [32]	CVLT-II	specific cognitive domains	—	CRT	CVLT-II	—	—
Andrade et al. (2022) [18]	ADAS-Cog	N/A	ADAS-Cog	—	—	—	—
Lu et al. (2019) [29]	ADAS-Cog, RT of N-back task	specific cognitive domains	ADAS-Cog	FDS	Logical memory tests	—	CVFT
Gonzalez et al. (2021) [30]	MoCA (Hong Kong), DS, TMT	RBMT-3	MoCA	FDS	RBMT-3	TMT-B	—
Inagawa et al. (2019) [33]	attrition rate of Kanji connection task	ADAS-Cog, MMSE, FAB, CDR-J	ADAS-Cog	—	—	FAB	—
Roncero et al. (2017) [34]	Spontaneous naming task	FDS, verbal fluency, MoCA, MMSE	—	TDS	—	—	accuracy on trained naming items
de Sousa et al. (2020) [35]	OLM-immediately after training	OLM-1 month delay after training	—	—	OLM	—	—
Cotelli et al. (2014) [28]	FNAT	Picture naming task, RBMT, BADA, RAVLT	MMSE	TMT-A	RAVLT, Delayed recall	TMT-B	Picture naming task
Brem et al. (2020) [36]	ADAS-Cog	ADCS-CGIC; ADCS-ADL	ADAS-Cog	—	—	—	—
Vecchio et al. (2022) [37]	ADAS-Cog	N/A	ADAS-Cog	—	—	—	—
Rabey et al. (2013) [38]	ADAS-Cog	CGIC	ADAS-Cog	—	—	—	—
Lee et al. (2016) [17]	ADAS-Cog	MMSE	ADAS-Cog	—	—	—	—
Bagattini et al. (2020) [39]	MMSE	specific cognitive domains	MMSE	TMT-A	RAVLT, delayed recall	—	PVF
Zhang et al. (2019) [40]	ADAS-Cog	ACE-III	ADAS-Cog	ACE-III-attention	ACE-III-memory	—	ACE-III-language

*MMSE* Mini-Mental State Examination, *FAB* Frontal Assessment Battery, *CDR-J* clinical dementia rating-Japanese version;CVLT-II, Total Learning- T score (age and education adjusted) on the California Verbal Learning Task, *CRT* Choice reaction time, *ADAS-Cog* Alzheimer Disease Assessment Scale, cognitive subscale, *DS* Digit span, *FDS* forward digit span, *CFVT* Category verbal fluency test, *TDS* Total digit span, *OLM* Object-Location Memory, *TOSL* Test of Strategic Learning, *DKEFS-CWI* Delis–Kaplan executive function system-word interference test, *FNAT* Face-name association memory task, *MoCA* Montreal Cognitive Assessment, *RBMT* Rivermead Behavioral Memory Test, *TMT* Trail Making Test, *BADA* Battery for Analysis of Aphasic Deficits, *RAVLT* Rey auditory verbal learning test, *CGIC* Clinical global impression of change, *PVF* Phonemic verbal fluency, *ACE-III* Addenbrooke’s Cognitive Examination III

#### Effects of NIBS combined with CT on different cognitive domains

**Fig. 4:**
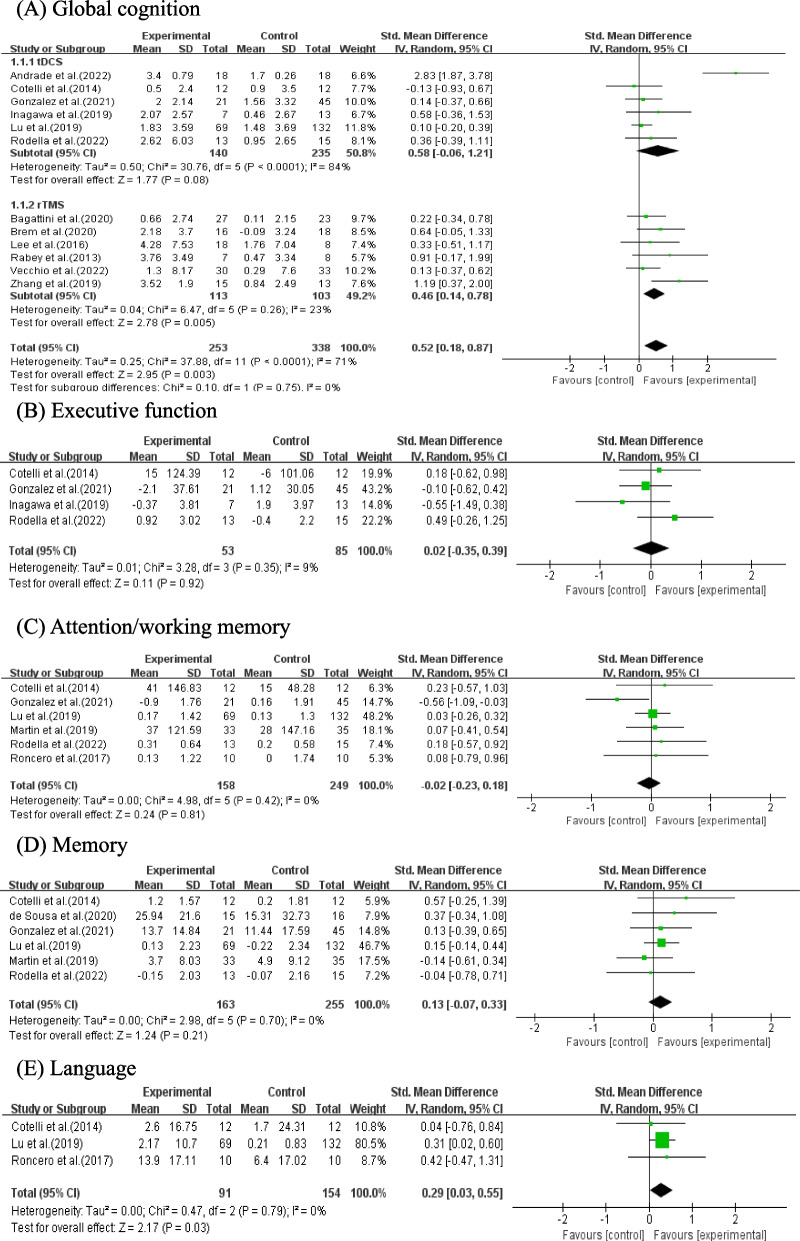
Meta-analysis of NIBS combined with CT on different cognitive domains (**A**-**E**)

Total of 12 studies with 591 patients reported global cognition scores including 6 studies performing tDCS combined with CT (*n* = 375) and 6 studies performing rTMS combined with CT (*n* = 216). The result of meta-analysis showed that NIBS combined with CT significantly improved global cognition scores in AD and MCI (SMD = 0.52, 95% CI (0.18, 0.87), *p* = 0.003; Fig. 4A). In subgroup data analyses, rTMS combined with CT significantly improved global cognition scores in AD and MCI (SMD = 0.46, 95% CI (0.14, 0.78), *p* = 0.005; Fig. 4A), while tDCS combined with CT showed no statistically significant effect on global cognition in AD and MCI (SMD = 0.58, 95% CI (-0.06, 1.21), *p* = 0.08; Fig. 4A).

For meta-analysis of specific cognitive domains, only studies involving tDCS combined with CT reported the results of specific cognitive domains scores. Three studies with 245 patients showed that tDCS combined with CT improved language scores compare to the control group (SMD = 0.29, 95% CI (0.03, 0.55), *p* = 0.03; Fig. 4E). However, the pooled results of 4 studies with 138 patients on execution function (SMD = 0.02, 95% CI (-0.35, 0.39), *p* = 0.92, Fig. 4B), 6 studies with 407 patients on attention/working memory (SMD = -0.02, 95% CI (-0.2, 0.18), *p* = 0.81, Fig. 4C), 6 studies with 418 patients on memory (SMD = 0.13, 95% CI (-0.07, 0.33), *p* = 0.21, Fig. 4D) all showed no statistically improvement.

#### Effects of NIBS combined with CT in patients with different diagnosis

**Fig. 5:**
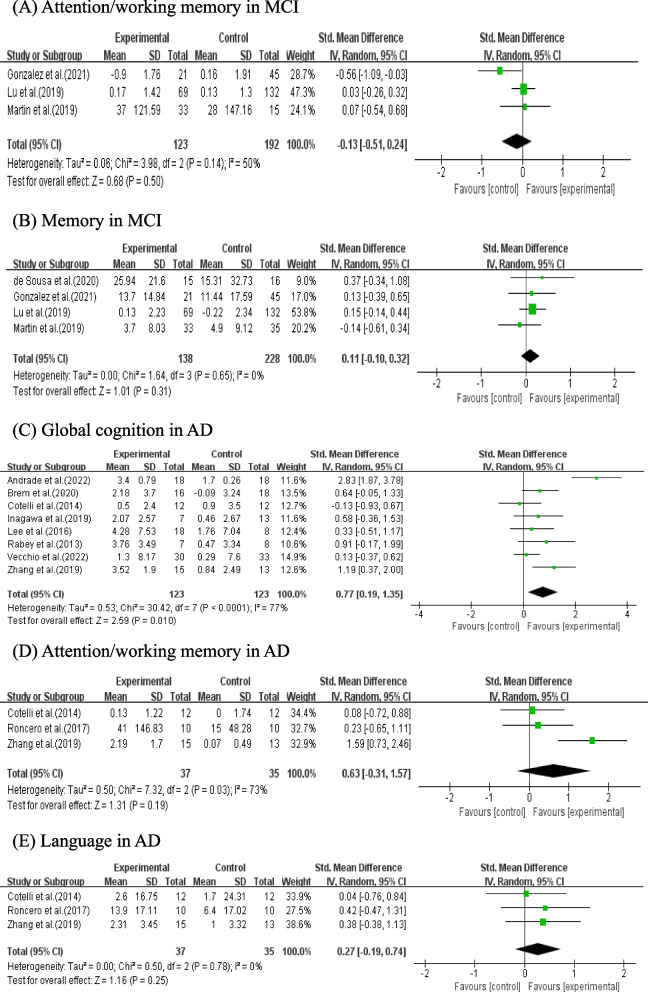
Meta-analysis of NIBS combined with CT on cognitive domains in patients with different diagnosis (**A**-**E**)

Three studies with 315 patients and 5 studies with 382 patients reported attention/working memory and memory scores in MCI, respectively. However, there was no statistically effect of NIBS combined with CT on attention/working memory (SMD = 0.13, 95% CI (-0.51, 0.24), *p* = 0.50; Fig. 5A) or memory scores (SMD = 0.11, 95% CI (-0.10, 0.32), *p* = 0.31; Fig. 5B).

Eight studies with 246 patients reported global cognition scores in AD. The result showed that NIBS combined with CT was statistically significant improvement on global cognition scores in AD (SMD = 0.77, 95% CI (0.19, 1.35), *p* = 0.01; Fig. 5C). However, the pooled results of 3 studies with 72 patients did not identify a statistically significant improve attention/working memory (SMD = 0.63, 95% CI (-0.31, 1.57), *p* = 0.19; Fig. 5D) or language scores (SMD = 0.27, 95% CI (-0.19, 0.74), *p* = 0.25; Fig. 5E) in AD.

#### Effects of NIBS combined with CT on follow-up

**Fig. 6:**
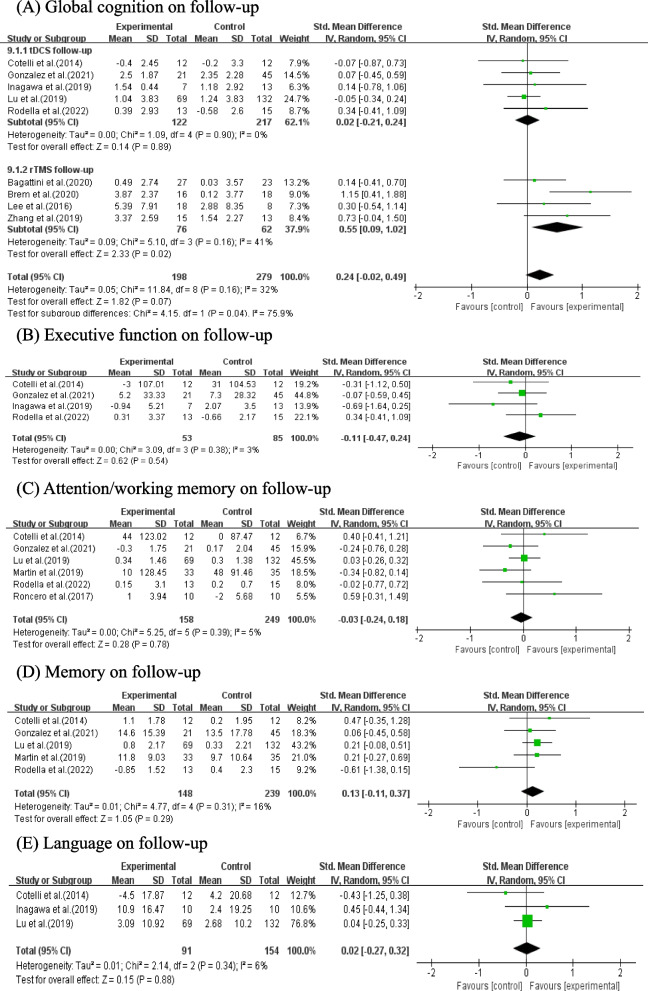
Meta-analysis of NIBS combined with CT on follow-up (**A**-**E**)

A total of 9 studies with 477 patients reported follow-up global cognition including 5 studies performing tDCS combined with CT (*n* = 339) and 4 studies performing rTMS combined with CT (*n* = 138). The result showed that there were no statistically global cognition improvement on follow-upin AD and MCI (SMD = 0.24, 95% CI (-0.02, 0.49), *p* = 0.07, Fig. 6A). While the result of subgroup analysis showed AD and MCI achieved signifcant follow-upglobal cognition improvement in rTMS combined with CT group (SMD = 0.55, 95% CI (0.09, 1.02), *p* = 0.02, Fig. 6A).

Furthermore, there were no statistically executive function improvement on follow-up in 4 studies with 138 patients (SMD = -0.30, 95% CI (-0.47, 0.24), *p* = 0.54, Fig. 6B), follow-up attention/working memory in 6 studies with 407 patients (SMD = -0.03, 95% CI (-0.24, 0.18), *p* = 0.78, Fig. 6C), follow-up memory in 5 studies with 387 patients (SMD = 0.13, 95% CI (-0.11, 0.37), *p* = 0.29, Fig. 6D) or follow-up language in 3 studies with 245 patients (SMD = 0.02, 95% CI (-0.27, 0.32), *p* = 0.88; Fig. 6E) either.

#### Effects of NIBS combined with CT in patients with different diagnosis on follow-up

**Fig. 7:**
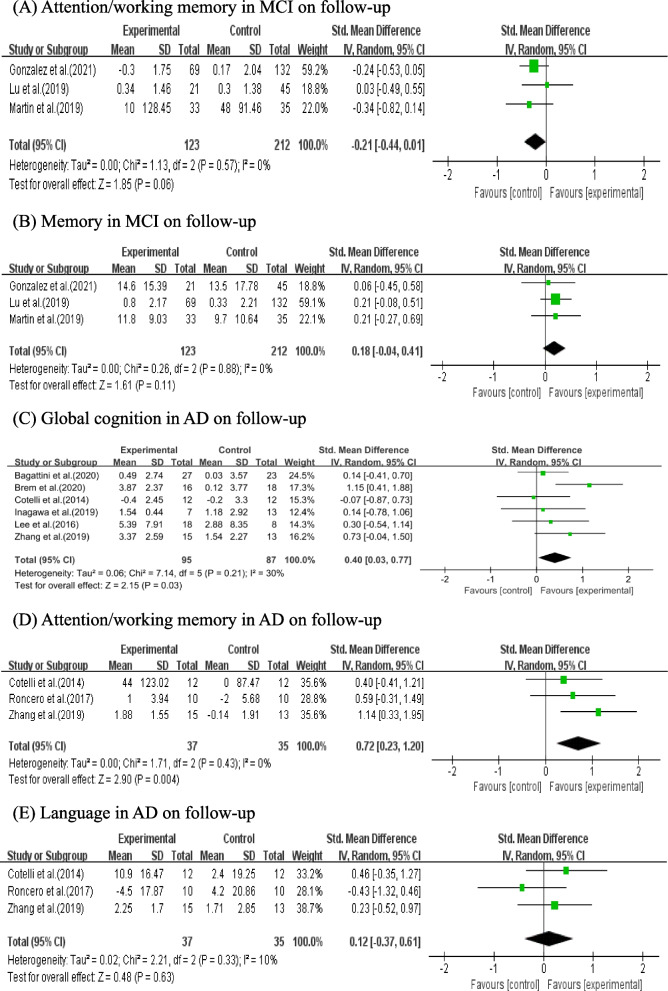
Meta-analysis of NIBS combined with CT in patients with different diagnosis on follow-up (**A**-**C**)

Three studies with 335 patients reported follow-up attention/working memory and follow-up memory scores in MCI. The pooled results showed that MCI did not achieved signifcant follow-up attention/working memory (SMD = -0.21, 95% CI (-0.44, 0.01), *p* = 0.06; Fig. 7A) or follow-up memory scores (SMD = 0.18, 95% CI (-0.04, 0.41), *p* = 0.11; Fig. 7B) improvement in NIBS combined with CT group.

Six studies with 182 patients and 3 studies with 72 patients reported follow-up global cognition and follow-up attention/working memory in AD, respectively. The pooled results showed NIBS combined with CT signifcantly improved follow-up global cognition (SMD = 0.40, 95% CI (0.03, 0.77), *p* = 0.03; Fig. 7C) and follow-up attention/working memory (SMD = 0.72, 95% CI (0.23, 1.20), *p* = 0.004; Fig. 7D) in AD. However, 3 studies with 72 patients did not achieve signifcant follow-up language improvement in AD (SMD = 0.12, 95% CI (-0.37, 0.61), *p* = 0.63; Fig. 7E).

## Discussion

This systematic review and meta-analysis aimed to evaluate the effects of NIBS combined with CT on cognitive function in AD and MCI including 15 studies with patients. The results of meta-analysis provided the following clear evidence: (1) rTMS combined with CT could improve short-term and follow-up global cognition in AD; (2) only AD could achieve short-term and follow-up global cognition improvement from NIBS combined with CT; (3) the benefits of NIBS combined with CT on follow-up attention/working memory were observed in AD; (4) tDCS combined with CT could improve short-term language in AD and MCI.

In this meta-analysis, we provided clear evidence that NIBS combined with CT could improve global cognition in AD and MCI as compared with only NIBS, CT or placebo. In addition, patients with AD achieved global cognition improvement from NIBS combined with CT group. Study outcomes from Chu et al. [41] and Wang et al. [42] were inconsistent with our results. There was a possible reason that the results by Chu et al. might be due to the limited number studies using NIBS combined with CT. Although AD have limited benefits derived from CT [43], NIBS seemed to help them maximize the benefits from CT as much as possible. It is currently thought that NIBS is able to induces and acquires brain's capacity for neuroenhancement [44], which may improve cognitive performance of patients. As a treatment approach to activate brain, CT could enhance functional network connectivity and functional efficiency of brain regions [45], and improved neuroplasticity of brain. When NIBS combined with CT, two treatments showed a synergistic effect presenting with greater neuroenhancement and neuroplasticity of brain, thereby strengthening cognitive performance in AD and MCI. It was noteworthy that the effects of individualised CT might only benefit in one specific cognitive domain, making it difficult to generalize to other specific cognitive domains [28]. Given the limited data available of included studies, we couldn't draw conclusions about the effect of NIBS combined with CT on improving global cognition in MCI. A meta-analysis by Xu et al. [46] found that NIBS could improve global cognition in MCI. If future more studies could obtain supports of sufficient data, a reciprocal synergistic effect of NIBS combined with CT in MCI maybe support causal hypothesis.

The result of subgroup analysis showed that rTMS combined with CT could improve global cognition in AD and MCI, while tDCS combined with CT not. Due to the absence of significantly effective pharmacotherapy or non-drug therapy on cognitive rehabilitation, patients and their families often struggle to choose which intervention would be more beneficial. Comparative efficacy of rTMS and tDCS in AD and MCI from previous studies was not clear [9, 47]. A meta-analysis by Wang et al. [42] did not compare the effects of rTMS combined with CT and tDCS combined with CT in AD and MCI. Our result contributed to providing recommendations for patients with cognitive impairment to choose more effective treatment of cognitive rehabilitation. Generally, rTMS produces more focused and deeper stimulations on brain regions and directly induces action potentials, whereas tDCS modulates the resting membrane potential of neurons and stimulates a more superficial and broader part of the cerebral cortex [48]. In addition, the current intensity of tDCS is more affected by skull and skin, resulting in some resistance to the current reaching the cerebral cortex. These influences weaken reciprocal synergistic effect between tDCS and CT, increasing treatment variability for patients. In studies involving rTMS combined with CT, only study by Bagattini et al. [39] included a small number of patients with MCI, therefore the meta-analysis results related to rTMS combined with CT might mainly reflect performances for patients with AD, not for patients with AD and MCI.

With regards to specific cognitive domain, tDCS combined with CT could improve language scores in AD and MCI, which is consistent with Chu et al. [41]. Meinzer et al. [49] recorded brain changes in MCI during tDCS stimulation using task-related and resting fMRI, showing that low accuracy of semantic flow tests might be related to hyperactivity of bilateral prefrontal area. The above study results found that Anodal tDCS signifcantly improved the accuracy of language tests in MCI, reduced task-related prefrontal hyperactivity and facilitated normalization of abnormal network structure in resting-state fMRI. The synergistic effects of tDCS combined with CT maybe enhance language improvement in AD and MCI. Nevertheless, as language function was measured only in 3 studies, and the main contribution of this result came from Lu et al. [29] with a risk of publication bias, the improvement of language should be taken with caution.

In follow-up cognition improvement, we found that NIBS combined with CT could improve follow-up global cognition in AD, especially for patients accepting rTMS combined with CT. The results indicated that NIBS combined with CT has a post-treatment sustainable effect in AD. Both NIBS and CT can regulate the excitability of neurons, alter neurotransmitter levels and enhance brain functional connectivity in AD and MCI [15, 50]. The synergistic effects of tDCS combined with CT maybe strengthen those brain excitability which may be related to sustainable effects [40]. Studies in this meta-analysis did not have a fixed follow-up period, with follow-up ranging from 2 weeks to 6 months. Moreover, follow-up effects could be influenced by multiple factors such as stimulation frequency, intensity, dropout rates and CT protocols [51], hence follow-up attention/working memory effects of NIBS combined with CT need to provide more evidences.

The strength of this article included the latest and most comprehensive synthesis of up-to-date evidence on the effects of NIBS combined with CT in AD and MCI. We registered in advance with a prespecified protocol on PROSPERO and strictly adhered to the PRISMA statement. The PEDro scale was used to assess methodological quality of included studies, and the Cochrane Handbook for Systematic Reviews of Interventions was used to evaluate the risk of bias. However, there were several limitations in this systematic review and meta-analysis. The use of different scales to evaluate global cognition and specific cognitive domains in AD and MCI might lead to high heterogeneity of the results. Some authors could not be contacted for raw data of three potentially eligible studies [52–54]. Due to the limited data available, cognitive effects of rTMS combined with CT on specific cognitive domain in AD and MCI could not be fully observed. It was also difficult to categorize patients into subgroups based on treatment parameters of NIBS and characteristics of CT, as these characteristics would lead to heterogeneity of some results.

## Conclusions

NIBS combined with CT, particularly rTMS combined with CT, has both short-term and follow-up effects on improving global cognition, mainly in patients with AD. tDCS combined with CT has advantages on improving language function in AD and MCI. Future more studies need evaluate cognitive effects of NIBS combined with CT on other specific cognitive domain in patients with cognitive deterioration.

### Supplementary Information

Supplementary Material 1.

Supplementary Material 2.

Supplementary Material 3.

## Data Availability

All data generated or analyzed are included in this published article and its supplementary materials.

## Abbreviations

AD: Alzheimer’s disease
MCI: Mild cognitive impairment
NIBS: Non-invasive brain stimulation
rTMS: Repetitive transcranial magnetic stimulation
tDCS: Transcranial direct current stimulation
CT: Cognitive training
