# Supplementary material for: The cognitive effect of non-invasive brain stimulation combined with cognitive training in Alzheimer’s disease and mild cognitive impairment: a systematic review and meta-analysis
Source: Alzheimers Res Ther. 2024 Jun 27;16:140. doi: 10.1186/s13195-024-01505-9 (PMC11212379; doi:10.1186/s13195-024-01505-9)
Supplement: Supplementary file 1 — Supplementary Material 1. [file 13195_2024_1505_MOESM1_ESM.docx]

Pubmed

((((((Mild cognitive impairment[MeSH Terms]) OR (MCI[Title/Abstract])) OR (Cognitive Dysfunction[Title/Abstract])) OR (cognitive impairment[Title/Abstract])) OR (early stage dementia[Title/Abstract])) OR (Alzheimer’s Disease[MeSH Terms])) OR (Dementia[MeSH Terms]) **AND** ((((((((Transcranial direct-current stimulation[MeSH Terms]) OR (tDCS[Title/Abstract])) OR (non-invasive brain stimulation[Title/Abstract])) OR (NIBS[Title/Abstract])) OR (neuromodulation[Title/Abstract])) OR (brain stimulation[Title/Abstract])) OR (Transcranial magnetic stimulation[MeSH Terms])) OR (Repetitive Transcranial magnetic stimulation[Title/Abstract])) OR (rTMS[Title/Abstract]) **AND** ((((((cognitive training[MeSH Terms]) OR (cognitive rehabilitation[Title/Abstract])) OR (cognitive intervention[Title/Abstract])) OR (training[Title/Abstract])) OR (memory training[Title/Abstract])) OR (executive function training[Title/Abstract])) OR (working memory training[Title/Abstract])

Web of science

((((((((TS=Mild cognitive impairment)) OR TI=(MCI)) OR TS=(Cognitive Dysfunction)) OR TI=(cognitive impairment)) OR TI=(early stage dementia)) OR TS=(Alzheimer’s Disease)) OR TS=(Dementia)) **AND** ((((((((TS=(Transcranial direct-current stimulation)) OR TI=(tDCS)) OR TI=(non-invasive brain stimulation)) OR TI=(NIBS)) OR TI=(neuromodulation)) OR TI=(brain stimulation)) OR TI=(rTMS)) OR TI=(Repetitive Transcranial magnetic stimulation)) OR TS=(Transcranial magnetic stimulation) **AND** ((((((TS=(cognitive training)) OR TI=(cognitive rehabilitation)) OR TI=(cognitive intervention)) OR TI=(memory training)) OR TI=(executive function training)) OR TI=(working memory training)) OR TI=(training)

Cochrane Library

MeSH descriptor: [Cognitive Dysfunction] explode all trees OR MeSH descriptor: [Alzheimer Disease] explode all trees OR MeSH descriptor: [Dementia] explode all trees OR (MCI):ti,ab,kw OR (cognitive impairment):ti,ab,kw OR (early stage dementia):ti,ab,kw **AND** (Transcranial direct-current stimulation):ti,ab,kw OR (tDCS):ti,ab,kw OR (non-invasive brain stimulation):ti,ab,kw OR (NIBS):ti,ab,kw OR (neuromodulation):ti,ab,kw OR (brain stimulation):ti,ab,kw OR (Transcranial magnetic stimulation):ti,ab,kw OR (Repetitive Transcranial magnetic stimulation):ti,ab,kw OR (rTMS):ti,ab,kw **AND** MeSH descriptor: [Cognitive Training] explode all trees OR (cognitive rehabilitation):ti,ab,kw OR (memory training):ti,ab,kw OR (executive function training):ti,ab,kw OR (working memory training):ti,ab,kw

EMBASE

'mild cognitive impairment'/exp OR 'cognitive dysfunction':ab,ti OR 'mci':ab,ti OR 'cognitive impairment':ab,ti OR 'early stage dementia':ab,ti OR 'alzheimer disease'/exp OR 'dementia'/exp **AND** 'transcranial direct current stimulation'/exp OR 'transcranial magnetic stimulation'/exp OR 'repetitive transcranial magnetic stimulation'/exp OR 'tdcs':ab,ti OR 'non-invasive brain stimulation':ab,ti OR 'nibs':ab,ti OR 'neuromodulation':ab,ti OR 'brain stimulation':ab,ti OR 'rtms':ab,ti **AND** 'cognitive rehabilitation'/exp OR 'cognitive training':ab,ti OR 'cognitive intervention':ab,ti OR 'memory training':ab,ti OR 'executive function training':ab,ti OR 'working memory training':ab,ti

EBSCO

TI Mild cognitive impairment OR TI MCI OR SU cognitive dysfunction OR TI cognitive impairment OR TI early stage dementia OR SU Alzheimer’s Disease OR SU Dementia OR AB Mild cognitive impairment OR AB MCI OR AB cognitive impairment OR AB early stage dementia **AND** SU transcranial direct current stimulation OR TI tDCS OR TI non-invasive brain stimulation OR TI NIBS OR TI neuromodulation OR TI brain stimulation OR SU Transcranial magnetic stimulation OR TI Repetitive Transcranial magnetic stimulation OR TI rTMS OR AB NIBS OR AB neuromodulation OR AB brain stimulation OR AB Repetitive Transcranial magnetic stimulation OR AB Rtms **AND** SU cognitive training OR TI cognitive rehabilitation OR TI cognitive intervention OR TI memory training OR TI executive function training OR TI working memory training OR TI training OR AB cognitive rehabilitation OR AB cognitive intervention OR AB memory training OR AB executive function training OR AB working memory training OR AB training AND
