# Supplementary material for: The cognitive effect of non-invasive brain stimulation combined with cognitive training in Alzheimer’s disease and mild cognitive impairment: a systematic review and meta-analysis
Source: Alzheimers Res Ther. 2024 Jun 27;16:140. doi: 10.1186/s13195-024-01505-9 (PMC11212379; doi:10.1186/s13195-024-01505-9)
Supplement: Supplementary file 3 — Supplementary Material 3. [file 13195_2024_1505_MOESM3_ESM.docx]

|  | our manuscript | Wang et al.[1] | Gonzalez et al.[2] | Chu et al.[3] | Burton et al.[4] | Teselink et al.[5] |
| --- | --- | --- | --- | --- | --- | --- |
| Search strategy | **Five databases** including PubMed, Web of Science, EBSCO, Cochrane Library and Embase were searched up from inception to 20 **November 2023**. | **Four databases** including PubMed, Web of Science, ProQuest and Central Cochrane library databases were searched up from inception to **December 2022**. | **Five databases** including PubMed, Web of Science, Science Direct, MEDLINE, and PsycINFO were searched up from inception to **November 2017**. | **Three databases** including PubMed, Embase and the Cochrane Library were searched up from inception **to 5 March 2020.** | **Five databases** including Ovid MEDLINE, PsycINFO, Scopus, CINAHL, and Cochrane Central were searched. | **Three databases** including MEDLINE, PsycINFO, and Embase were searched up from inception to **June 2021**. |
| Inclusion criteria | Study design was randomized controlled trial or randomized cross-over design published. | Study design was randomized controlled trial. | 1.Participants included in study were older adults with MCI and persons with **a diagnosis of dementia**.  2.The comparison group included a placebo with **sham tDCS,** **sham tDCS combined with CT.** | 1.a component **network meta-analysis**;  2. The author included studies of **only NIBS** or studies of NIBS combined with CT. | 1. focused on a neuropsychiatric Population with **primary psychiatric disorders** or neurodegenerative conditions. | Studies must have administered multi-session non-invasive brain stimulation including **tDCS, TMS, or rTMS.** |
| Number of included studies | We included 15 studies with 685 patients. | The author included 12 studies with 587 AD patients. | The author included 16 studies **but only 5 studies on tDCS combined with CT.** | The author included 27 studies **but only 8 studies on NIBS combined with CT**. | The author included 15 studies but **only 7 studies in MCI or AD**. | The author included 19 studies **but only 4 studies on NIBS combined with CT**. |
| Quality assessment | we used both the PEDro scale and the revised Cochrane risk of bias tool in our study, because each tool offers unique advantages and perspectives in assessing the quality and risk of bias in clinical trials. **The PEDro scale** is specifically designed for physiotherapy research and provides a numerical rating, while **the Cochrane risk of bias tool** is applicable to a broader range of clinical trials and evaluates risk levels in five distinct domains. By utilizing both tools, we aimed to ensure a comprehensive and robust assessment of the included studies, accounting for various aspects of trial quality and bias risk | The author assessed the methodological quality using the revised *Cochrane* risk of bias tool. | The author assessed the methodological quality using the revised Cochrane risk of bias tool. | The author assessed the methodological quality using the revised Cochrane risk of bias tool. | The author assessed the methodological quality using the revised Cochrane risk of bias tool. | The author assessed the methodological quality using the revised Cochrane risk of bias tool and Newcastle-Ottawa Scale. |
| Meta-analysis strategy | 1. We observed the effects of NIBS combined with CT on different cognitive domains (global cognition, executive function, attention/working memory, memory, and language);  2. We observed the effects of NIBS combined with CT in patients with different diagnosis (AD and MCI);  3. We observed the effects of NIBS combined with CT on follow-up (different cognitive domains and patients with different diagnosis);  4. **In subgroup analysis, we compared the effects of rTMS combined with CT and tDCS combined with CT on global cognition in AD and MCI.** | 1. The author only observed the effects of NIBS combined with CT on global cognition and short time memory;  2. The author only observed the follow-up effects of NIBS combined with CT on long time memory. | There is **no conclusive advantage** in coupling tDCS with cognitive training. | 1.The author observed the effects of NIBS on different cognitive domains (global cognition, memory, and verbal fluency, Working memory, executive function);  2. The author observed long-lasting effects of NIBS after 1 month. | 1. The author observed the effects of NIBS combined with CT on different cognitive domains **in neuropsychiatric disorders** (global cognition, executive function, attention/working memory, memory, and language); | 1. The author observed the effects of NIBS on global cognition and neuropsychiatric symptoms. |
| Primary outcome | In addition to the effects of NIBS combined with CT in AD and MCI (SMD=0.52, 95% CI (0.18, 0.87), p=0.003), we emphasized the effects of **rTMS combined with CT on global cognition in AD and MCI** (SMD=0.46, 95% CI (0.14, 0.78), p=0.005), **tDCS combined with CT on language function in AD and MCI** (SMD=0.29, 95% CI (0.03, 0.55), p=0.03), and **rTMS combined with CT on follow-up global cognition in AD and MCI** (SMD=0.55, 95% CI (0.09, 1.02), p=0.02). | Wang et al. (2024) indicated a positive effect of NIBS-CT on overall cognitive function (SMD = -0.52, 95%CI (-0.93, -0.11)) and ADAS-COG (MD=-1.16, 95%CI (-1.69, -0.63)) but not on memory. | There is **no conclusive advantage** in coupling tDCS with cognitive training. | 1.Combining CT with HFrTMS (SMD=-0.15, 95% CI (-0.19, 0.89)) and atDCS (SMD=-0.67, 95% CI (-2.06, 0.72)) on global cognition **did not result in larger effects;**  2. Long-lasting effects at 1-month follow-up for HFrTMS+CT (SMD=0.68, 95% CI (-1.18, 2.54)) and atDCS (SMD=-0.76, 95% CI (-3.09, 1.56)) **did not reach statistical significance**.  3. Combining CT with HFrTMS and atDCS on short-term and long-lasting memory function **did not significantly increase the effect;**  4. Combining CT with atDCS on short-term verbal fluency **had a larger effect** (SMD=0.49, 95% CI (0.03, 0.95));  5. HFrTMS+CT showed **statistically significant effects** at 1-month follow-up (SMD=0.93, 95% CI (0.15, 1.70)) | Burton et al. indicated a positive effect of attention/working memory for all studies combined (SMD = 0.26; 95% CI, 0.06 to 0.47; z = 2.57; p = .01) **but not for neurodegenerative disorders** (SMD = 0.10; 95% CI, 20.14 to 0.35; z = 0.83; p = .41). | A subgroup analysis based on the administration of NIBS combined with CT revealed that patients who received CT **did not significantly improve** on global cognition (SMD= 0.295; 95% CI= − 0.38, 0.97; p = 0.393), whereas those who received NIBS only improved on global cognition (SMD= 1.389; 95% CI= 0.578, 2.2; p = 0.019). |

1. Wang JY, Qin JY, Ye JY, Li WT, Tong MQ, Ouyang H, et al. The Therapeutic Effects of Noninvasive Brain Stimulation Combined with Cognitive Training in Elders with Alzheimer's Disease or Amnesic Mild Cognitive Impairment. The journal of prevention of Alzheimer's disease. 2024;11:222-9. <https://doi.org/10.14283/jpad.2024.1>.

2. Cruz Gonzalez P, Fong KNK, Chung RCK, Ting KH, Law LLF, Brown T. Can Transcranial Direct-Current Stimulation Alone or Combined With Cognitive Training Be Used as a Clinical Intervention to Improve Cognitive Functioning in Persons With Mild Cognitive Impairment and Dementia? A Systematic Review and Meta-Analysis. Front Hum Neurosci. 2018;12:416. <https://doi.org/10.3389/fnhum.2018.00416>.

3. Chu CS, Li CT, Brunoni AR, Yang FC, Tseng PT, Tu YK, et al. Cognitive effects and acceptability of non-invasive brain stimulation on Alzheimer's disease and mild cognitive impairment: a component network meta-analysis. Journal of neurology, neurosurgery, and psychiatry. 2021;92:195-203. <https://doi.org/10.1136/jnnp-2020-323870>.

4. Burton CZ, Garnett EO, Capellari E, Chang SE, Tso IF, Hampstead BM, et al. Combined Cognitive Training and Transcranial Direct Current Stimulation in Neuropsychiatric Disorders: A Systematic Review and Meta-analysis. Biological psychiatry Cognitive neuroscience and neuroimaging. 2023;8:151-61. <https://doi.org/10.1016/j.bpsc.2022.09.014>.

5. Teselink J, Bawa KK, Koo GK, Sankhe K, Liu CS, Rapoport M, et al. Efficacy of non-invasive brain stimulation on global cognition and neuropsychiatric symptoms in Alzheimer's disease and mild cognitive impairment: A meta-analysis and systematic review. Ageing Res Rev. 2021;72:101499. <https://doi.org/10.1016/j.arr.2021.101499>.
